# Preparation of Nanocomposite Alginate Fibers Modified with Titanium Dioxide and Zinc Oxide

**DOI:** 10.3390/polym12051040

**Published:** 2020-05-02

**Authors:** Dominik Borkowski, Izabella Krucińska, Zbigniew Draczyński

**Affiliations:** 1 Institute of Textile Materials and Polymer Composites, Lodz University of Technology, Żeromskiego 116, 90-924 Lodz, Poland; dominik.borkowski@dokt.p.lodz.pl (D.B.); izabella.krucinska@p.lodz.pl (I.K.); 2 Lukasiewicz Research Network - Institute of Biopolymers and Chemical Fibers, Skłodowskiej-Curie 19/27, 90-570 Lodz, Poland

**Keywords:** alginate fibers, titanium (IV) oxide, zinc oxide, FTIR, WAXS, sorption, retention, antimicrobial activity testing

## Abstract

Active dressings based on natural polymers are becoming increasingly popular on the market. One of such polymers is alginate, which is characterized by biodegradability, resorbability, has no carcinogenic properties, does not have allergenic or hemostatic properties, and has a confirmed lack of toxicity. However, this polymer does not show biocidal and biostatic properties, therefore the purpose of this research was to select the appropriate conditions for the production of calcium alginate fibers modified with nano titanium dioxide and nano zinc oxide. It was assumed that the presence of nano metal oxide fillers will give antibacterial properties to formed fibers, which were used to form nonwovens. The following article presents a comparative analysis of nonwovens made of alginate fibers, without nano additives, with nonwovens made of alginate fibers containing in their structure 7% titanium dioxide and nonwovens made of alginate fibers containing 2% ZnO. The selection of the nano additive content was determined by the spinning ability of the developed polymer solutions. Based on the results contained in the article, it was found that the introduction of modifiers in the structure of fibers increases the diameter of the fiber pores, which improves the sorption and retention properties of the obtained fibers, and also gives differentiated antibacterial properties to the obtained nonwovens depending on the type of nano additive used. Greater activity against Escherichia coli, Staphylococcus aureus strains and Aspergillus Niger molds was shown in nonwovens made of 2% ZnO modified fibers compared to nonwovens made from TiO_2_ modified fibers.

## 1. Introduction

Recent years have shown that biomedical engineering and nanotechnology are the most evolving scientific disciplines [1]. Nanotechnology and biomedicine allow us to receive increasingly new composite materials that are beginning to play a significant role in medicine.

Biomedical engineering as a science uses natural polysaccharides such as chitosan, chitin, alginate and their derivatives [2,3,4,5]. These polysaccharides are used, among others, in dressings for the treatment of wounds that are difficult to heal [6] and as scaffolds for cell culture [7]. The use of these natural polymers in biomedicine is associated with their specific chemical, physical and biological properties, which include biodegradability, membrane and fiber-forming abilities, bioactivity and biocompatibility [1]. 

Nanotechnology is a rapidly developing field which makes it possible to obtain and test materials that have at least one dimension below 100 nm [8]. Thanks to this science, we can create new and better products that have improved chemical, physical and biological properties [9]. Combining it with biomedical engineering means that the obtained dressings gain better properties, accelerate tissue regeneration, which results in faster wound healing [6]. 

For the creation of such dressings, as already mentioned, among others, a polysaccharide is used, which is alginate. Alginate is most often obtained from marine algae. The main algae from which alginate is obtained are brown algae (Phaeophyceae) and keratosis (Rhodophyta) [10,11]. Alginates have a linear structure of macromolecules, which is made up of two types of mer. These mers are derived from α-L-guluronic acid (G) residues and β-D-mannuronic acid (M) residues and are linked by a β-1-4 glycosidic linkage. The G and M units present in the alginate polymer chain can form three types of segments. These are MM, GG and MG blocks. Alginates have many properties that are important for their medical purpose. These properties include, among others: rapid degradation under the influence of moisture at an elevated temperature, biodegradability, resorbability, not causing an allergic reaction, demonstrating hemostasis, nontoxicity, non-carcinogenicity [12,13,14]. Due to such specialized properties, alginates have found many applications in medicine and pharmacy. One of them is the use of this polymer for the construction of new biosensors [15,16]. Tissue engineering is another application of alginates [17]. Alginates have many applications in supporting regeneration processes ofbone [18], skin [19], heart muscle [20], cartilage [21,22], liver [6], and now even in nerve regeneration [23]. However, the main and first use of alginates was to use them for the production of dressing materials (gases, bandages, filters). The main advantage of using these dressings that are based on calcium or sodium alginate is that they do not stick to the wound [24]. Dressings made of alginate fibers are classified as active dressings. They are used for exudative, bleeding wounds and for various types of burns and open fractures, as well as for places that are sensitive to pain [17].

Thanks to the continuous development of biomedicine and nanotechnology, it is possible to modify previously known fiber properties. Alginate fibers are a susceptible raw material for various modifications. To improve their regenerative capabilities, thanks to which the whole process of wound healing will be accelerated, they can be modified with various nano additives [25]. One such nano additive is titanium dioxide and zinc oxide.

Titanium dioxide is a white and gray odorless solid [26], occurring in nature in three different crystal structures. These are anatase, rutile and brukite [27]. The most important properties of this compound are, among others, environmental neutrality, high chemical resistance, nontoxicity, biocompatibility, hydrophilicity, photocatalytic and antibacterial properties [28,29]. Thanks to these features, the oxide has found many applications in medicine and dentistry. One of them is the presence of titanium oxide in preparations for disinfecting surfaces, catheters or surgical instruments. Another application of TiO_2_ is tissue engineering, in which it can act as one of the components of biocomposites to destroy bacteria [30]. In cancer therapies, TiO_2_ nanoparticles are slowly used in the fight against cancers, e.g., glioma, because it has been proven that, under the influence of ultrasound and UV radiation, they destroy diseased cells [31]. Nano-TiO_2_ is also used in the reconstruction of facial tissues, as well as opaque in silicones, which affect the color behavior and the mechanical properties of materials [32]. Dentistry is another area where TiO_2_ is used, where titanium oxides are used in dental implants, thanks to which they affect implant durability, accelerate wound healing and affect faster bone growth [33,34].

The second compound that can modify the properties of alginate fibers is zinc oxide. ZnO is a semiconductor white solid belonging to the group II-VI of the periodic table, characterized by an energy gap, which at room temperature is 3.37 eV and increases with decreasing size of nanoparticles [35,36,37]. Zinc oxide is obtained by two methods, by roasting zinc ores or as a result of the zinc vapor combustion reaction [35]. Zinc oxide is found in various crystallographic systems such as zinc diaphragm, wurtzite [35,36,37,38]. ZnO is biosafe and biocompatible, which is its important advantage, and is also characterized by high antibacterial activity and drying properties [39]. Due to its anti-inflammatory and drying properties, zinc oxide is a component of UV protection ointments and creams containing micro- or nanoparticles of this compound [35]. One of the main uses of ZnO is to use it as an effective drug delivery system for various diseases [37]. Another application of this compound is its use in textile materials with unique physicochemical properties to neutralize and destroy bacteria that can grow on medical dressings during wound healing [40,41,42,43]. The presented literature review indicates the legitimacy of undertaking work on combining the health-promoting features of alginates and the listed nanocomponents.

The research presented in the article was aimed at determining the appropriate conditions for the production of calcium alginate fibers containing maximum amounts of titanium dioxide and zinc oxide nanofillers. As part of the work, the influence of the spun ratio and stretching on the structure and properties of formed fibers was determined.

## 2. Materials and Methods

### 2.1. Materials

To obtain nanocomposite fibers, sodium alginate PROTANAL LF 10/60LS from FMC Biopolymer (Philadelphia, PA, USA)with a molecular weight of 89 kg/mol and distilled water as a solvent was used. The nano additives introduced were nano titanium dioxide from Sigma Aldrich (Warsaw, Poland), with a grain size of less than 100 nm, as well as nano zinc from Sigma Aldrich (Warsaw, Poland), with grain size less than 100 nm. Alginate fibers were formed using the wet method from solution using a 500-hole nozzle with a hole diameter of 0.08 mm. Three solutions containing 14.8 g of polymer in 185.8 g of water were prepared for forming the fibers. The first solution contained no nano additives, 1.036 g of nano TiO_2_ (7% titanium dioxide by weight) was added to the second solution, while the third solution contained 0.296 g ZnO (2% zinc oxide by weight). The number of nano additives added was the maximum amount of fillers present in the solution, ensuring the ability to spin fibers from these solutions. The above content of introduced nano fillers was determined on the basis of the results of our previous tests (not presented in this paper). The resulting solutions were then mechanically mixed at 500 rpm for six hours and allowed to vent for 24 hours. An exemplary procedure for forming fibers from a wet solution is as follows. The spinning fluid was fed under a pressure of 3 atm to a gear pump with a capacity of 0.6 cm^3^/min. The fibers were formed using a 500-hole nozzle with a hole diameter of 0.08 mm with a feeding speed of 2 m/min. Then, the fibers were taken on the first set of galettes at a speed of 4.4 m/min (120% of spun ratio) and then stretched and picked up by the second set of galettes at a speed of 6.8 m/min (55 of stretching). The construction of the spinner enabled the stabilization of technological parameters at the assumed level. The whole fiber solidification process was carried out in two baths. The first bath—the solidifying bath—contained 3% aqueous CaCl_2_ solution at 25 °C, while the second bath—the plasticizing bath—contained 2% aqueous CaCl_2_ solution and had a temperature of 67 °C. The solidifying and plasticizing baths were in continuous circulation, the coagulation bath being fed concurrent to the solidified fiber, while the plasticizing bath was being fed counter current. After the stretching process, the fibers were collected continuously in the form of a bobbin winding. After the forming process, the fibers were kept in distilled water for another 24 hours. Then the fibers were dried at a temperature of about 25 °C under isometric conditions.

### 2.2. Methods

In order to assess the properties of the fibers produced, the following test methodology was applied, referring to both the chemical and supermolecular structure and the resulting physical properties of nanocomposite fibers. 

The chemical structure of the obtained fibers was confirmed by the FTIR analysis. The JASCO V-57PC FTIR NIR spectrophotometer (JASCO Deutschland GmbH, Pfungstadt, Germany) with an ATR adapter was used. The obtained spectra were the mean of 64 scans with a resolution of 4 cm^−1^.

The degree of fiber crystallinity and the size of the crystallinity areas were determined by means of wideangle differential diffractometry. The X-ray diffractometer (XRD/WAXS, Almelo, Netherlands) X’Pert PRO by PANalytical with CuKα source (λ = 0.154 nm) and the following parameters: accelerating voltage of 40 kV and anode current density of 30 mA. A semiconductor counter X’Celector (PANalytical, Almelo, Netherlands) was used as the detector. The diffraction patterns for the powdered samples were recorded over a 2θ range of 8°–58° with a step size of 0.015°.

The JSM-5200LV scanning electron microscope (JOEL, Warsaw, Poland) and sprayer JFC-1200 (JOEL, Warsaw, Poland) were used for macroscopic structure analysing. 

The thermal properties of the obtained fibers were examined with the SDT 2960 TA Instruments apparatus (Mettler-Toledo, Warsaw, Poland). The measurements were carried out in the range from −80 to 300 °C. The heating rate was 10 °C/min, while the cooling rate was 20 °C/min.

The porosity of the fibers was determined using the Micromeritics’ AutoPore IV 9500 Series (Kunash Instruments, Thane, India). 

The mechanical properties of fibers were determined using an INSTRON - type ripper (LaborTech, Auschwitz, Poland) in accordance with PN-EN ISO 5079:1999. 

Sorption was determined at 100% relative humidity and 20 °C. Water Retention Value was also determined using a laboratory centrifuge that enabled mechanical removal of water from fibers in the centrifugation process with an acceleration of 10,000 m/s^2^. The retention value was determined as the ratio of the weight of water remaining in the fiber after centrifugation to the weight of the completely dried fiber. 

Antimicrobial activity tests were performed using the AATCC 100-1998 “Antimicrobial Finishes of Textile Materials” method.

## 3. Results and Discussion

### 3.1. Infrared Spectroscopy—FTIR

**Figure 1:**
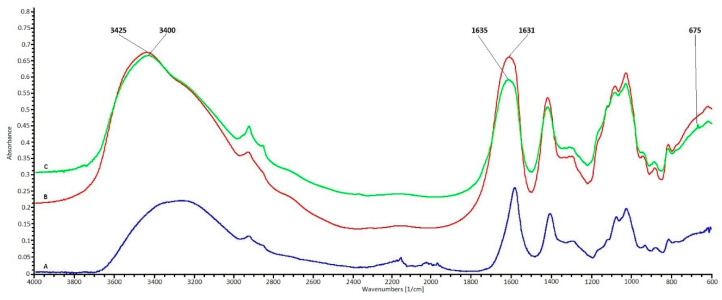
FTIR spectrum of alginate fibers. (**A**) Fibers without nano additive, (**B**) fibers with 2% nano additive ZnO, and (**C**) fibers with 7% nano additive TiO_2_.

FTIR spectroscopy analysis was used to confirm the presence of nano metal oxides in the fiber polymer matrix. The changes occurring in the IR spectra of fiber samples with nano metal oxides compared to the fiber spectrum without nano metal oxides were the basis for confirming their presence in the polymer matrix (Figure 1).

Comparing the FTIR ATR spectra of alginate fibers with and without nano fillers, changes suggesting their presence in the fiber polymer matrix can be observed. Analyzing the characteristic range for hydroxyl groups with a maximum of about 3400 cm^−1^, a signal widening is observed in the spectrum of fibers containing nano additives compared to the spectrum of fibers without nano additives. This phenomenon is probably the result of intermolecular interactions between hydroxyl groups of the polymer matrix and nano filler metal oxides. Similar signal broadening is observed for peaks characteristic of carboxyl groups with a maximum at 1590 cm^−1^ for the spectrum of fibers without nano additives. In the spectra of fibers containing nano additives, these signals are broadened and shifted towards the maximum at 1635 cm^−1^. The presence of a free electron pair on an oxygen atom in metal oxides can result in the formation of hydrogen bonds with hydroxyl and carboxyl groups present in the alginate chain. The signals in the range of FTIR ATR fibers shown in the 2000–2300 cm^−1^ range are derived from the germanium crystal ATR adapter and are not signals of the polymer matrix of the fibers. The appearance of peaks in the range below 700 cm^−1^ on the spectra of alginate fibers containing metal nano oxides (Figure 1B,C), absent in the spectrum of the fibers without additives (Figure 1A), indicates their presence in the polymer matrix. In the spectrum of fibers containing 7% TiO_2_, there is a characteristic narrow peak with a maximum at 675 cm^−1^ corresponding to bonding vibration in metal oxide (TiO_2_). Low ZnO concentration (2%) in the polymer matrix results in the lack of a clear appearance of the corresponding metal oxide signal. Comparative analysis of the FTIR ATR spectra of alginate fibers containing metal oxides with the spectrum of alginate fibers without the addition of metal oxides indicates the occurrence of intermolecular interactions confirming their presence in the fiber matrix.

### 3.2. Degree of Crystallinity—WAXS

**Figure 2:**
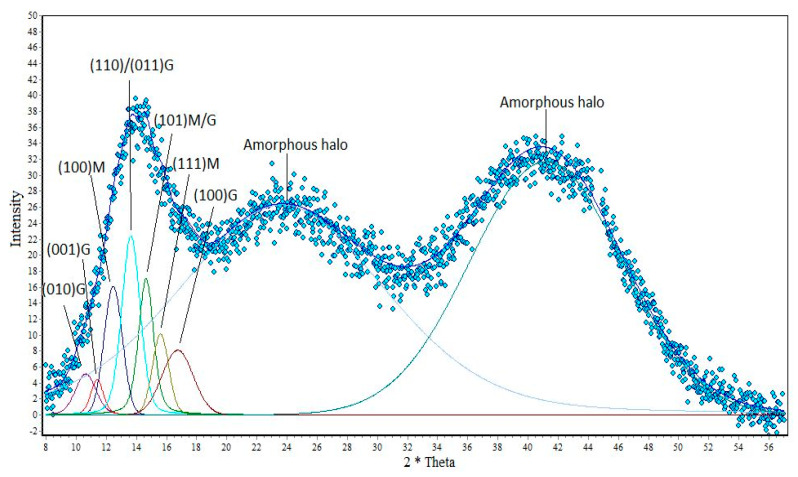
Distribution of the diffraction curve of calcium alginate fibers without nano additive.

**Figure 3:**
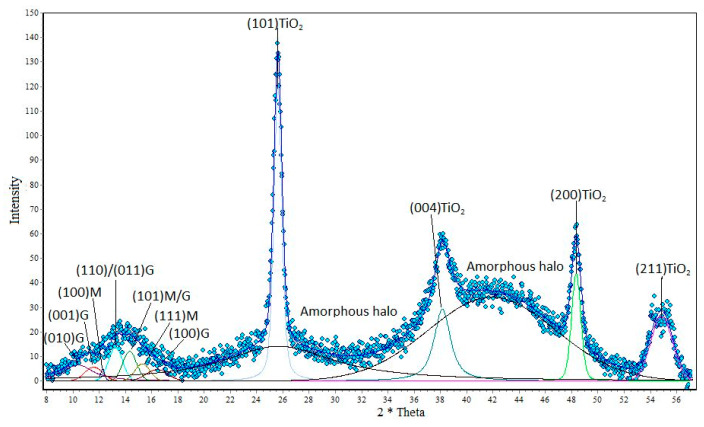
Distribution of the diffraction curve of calcium alginate fibers containing 7% titanium dioxide.

**Figure 4:**
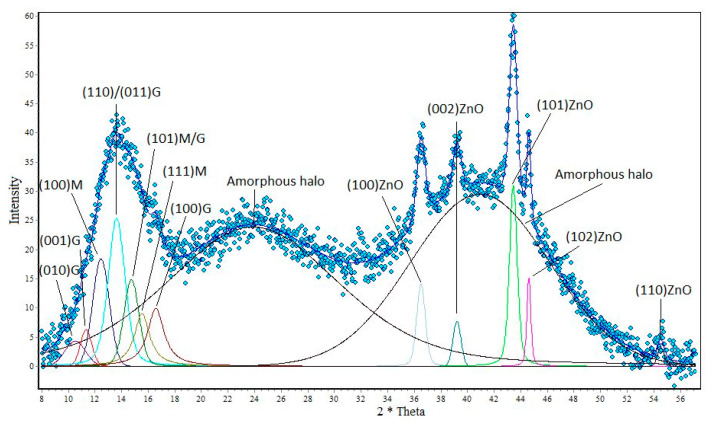
Distribution of the diffraction curve of calcium alginate fibers containing 2% ZnO.

In subsequent studies, the obtained research material was analyzed using the WAXS technique. Two parameters were determined by means of wide-angle X-ray diffraction: the degree of crystallinity and the size of the crystalline. The results are shown on the following diffractometers (Figure 2, Figure 3 and Figure 4).

Analyzing the graph (Figure 2) showing alginate fibers without a nano additive, you can see crystal peaks that are visible at 2θ angles from 10° to 17° and amorphous fields located on the graph at values of about 2θ = 24°, and also at 2θ = 41°. The next chart shows that with the appearance of nano additive, new peaks are formed in the fiber structures that are characteristic of titanium dioxide and zinc oxide. Analyzing the graph (Figure 3), it can be seen that the amorphous peaks decrease their intensity, and above them appear quite distinct crystalline peaks from titanium dioxide. These peaks have their values at angles of 2θ = 25.7°, 2θ = 35.6°, 2θ = 48.3° and 2θ = 55°. A similar relationship can also be seen for the graph showing fibers containing ZnO in their structure (Figure 4). On the WAXS distribution curve of alginate fibers containing ZnO, the amorphous peak also reduces its intensity and there are crystal peaks at angles of 2θ = 36.5°, 2θ = 39.2°, 2θ = 43.4°, 2θ = 44.6° and 2θ = 54.3°. The presence of the mentioned peaks confirms the presence of nano additives in the polymer matrix, while the decrease in the intensity of the amorphous peaks in the WAXS distribution curves of the fibers with modifiers indicates an increasing degree of crystallinity of the formed fibers. The degree of crystallinity was determined by the formula:

(1) $$x_{k}=\frac{A_{k}}{A_{k}+A_{a}}*100\%$$

where, *x*_k_—crystalline degree; *A*_k_—sum of crystal peak areas; *A*_a_—sum of amorphous peak areas.

Analyzing the results of Table 1, we can notice that the presence of nano additives in fiber structures increases their degree of crystallinity. An increase in the degree of crystallinity can be associated with the nucleation effect of the crystalline phase on TiO_2_ and ZnO particles. This effect affects the arrangement of the supermolecular structure of the polymer matrix of fibers, which increases their mechanical strength.

### 3.3. Scanning Electron Microscopy—SEM

**Figure 5:**
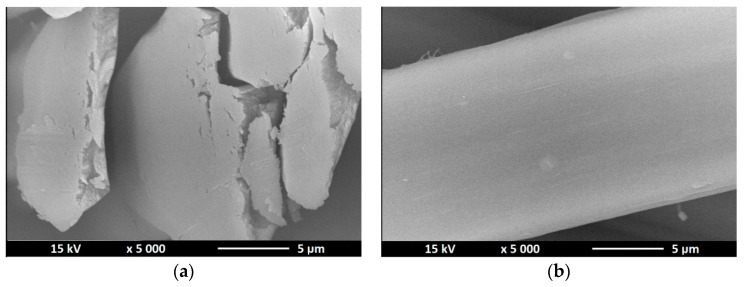
SEM images of cross section (**a**) and surface (**b**) of alginate fibers without nano additive.

**Figure 6:**
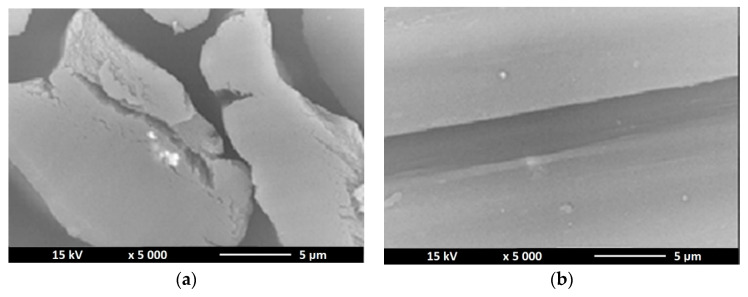
SEM images of cross section (**a**) and surface (**b**) of alginate fibers with 2% ZnO nano additive.

**Figure 7:**
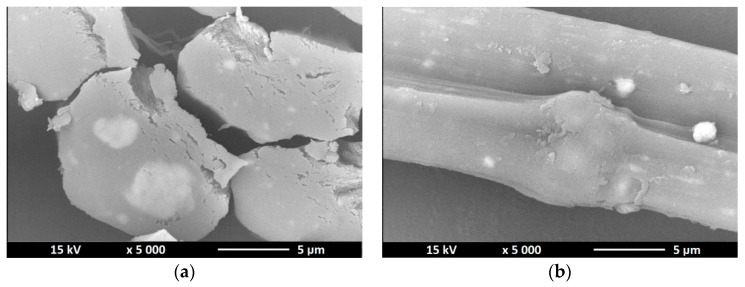
SEM images of cross section (**a**) and surface (**b**) of alginate fibers with a 7% TiO_2_ nano additive.

As part of confirming the presence of nano additives in the structure of nanocomposite fibers, a study was conducted consisting in observation of the microscopic structure of the obtained fibers. The pictures (Figure 5, Figure 6 and Figure 7) below show the cross section as well as surface of the obtained fibers.

SEM images show the presence of bright dots inside the fiber structure and on their surface. Any thickening of the surface or the appearance of bright dots on it also means the appearance of titanium dioxide or zinc oxide in a given place. For the fibers containing titanium dioxide in their structure, it can be seen that the solidification bath used had too low coagulation capacity, because the fiber cross section reveals the process of “gluing” elementary fibers. For fibers without a modifier, the surface was characterized by a fairly low defect, in contrast to fibers that had a nano additive in their structure. The nano additive located on the surface of the fibers and in cross sections is arranged in a chaotic and uneven manner. Comparing TiO_2_ modified fibers with fibers containing ZnO in their structure, we can see that in the case of the former we see a much larger amount of nanodrug in the fiber structure due to the higher concentration of titanium oxide in the spinning solution, which may be the reason that they occur in much larger clusters (agglomerates) than in the case of fibers containing zinc oxide in their structure.

### 3.4. Differential Scanning Calorimetry—DSC

**Figure 8:**
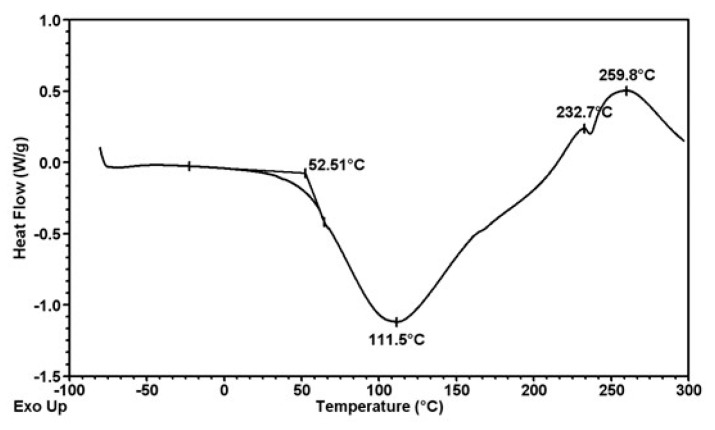
Thermogram of calcium alginate fiber without nano additive.

**Figure 9:**
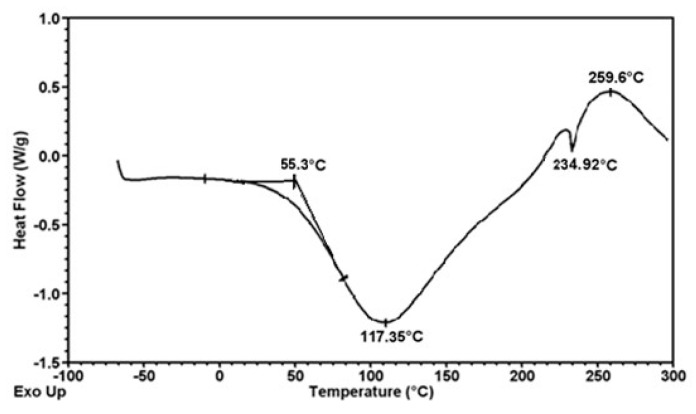
Thermogram of calcium alginate fiber with 2% nano Zn.

**Figure 10:**
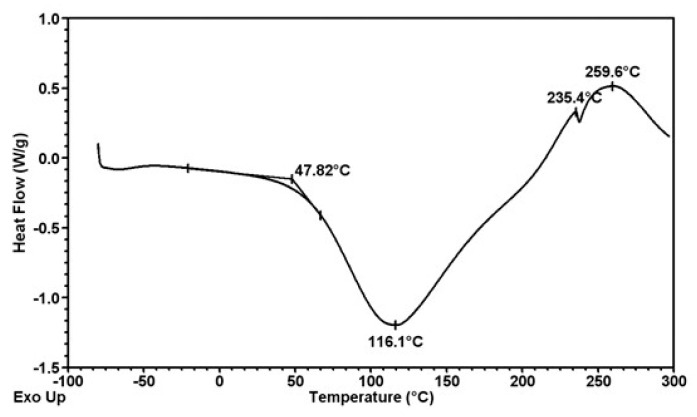
Thermogram of calcium alginate fiber with a 7% addition of titanium dioxide.

Thermal analysis of formed fibers was carried out by SDT 2960 TA Instruments. The graphs (Figure 8, Figure 9 and Figure 10) shown depict phase swaps of fibers under the heat.

Thermal analysis of the obtained fibers confirms that their thermal properties are not dependent on the presence of the modifier and its amount in the fiber matrix. In the analyzed cases, the change in the nature of the fiber material ends at a temperature of about 260 °C, regardless of the presence of titanium oxide or zinc oxide. Thermal decomposition takes place in several stages. The first of these is associated with the desorption of water molecules (endothermic effect on the DSC curve) and occurs from a temperature of 25 to 111 °C for fibers without nanoparticles (Figure 8), up to 116 °C for fibers with ZnO (Figure 9) and up to 117 °C for TiO_2_ (Figure 10). Another typical step for polysaccharides is a gradual exothermic effect in a temperature range of about 111–117 to 235 °C. This process may be associated with the occurring dehydration, as well as the breakdown of C=O and C–C bonds of the alginate chain. In addition, carbon structures are formed as a result of the pyrolysis process. The last stage of decomposition is a very strong exothermic effect visible on the DSC curve with a maximum at a temperature of about 260 °C. It is associated with progressive carbonization of polysaccharide fiber samples. The observed endothermic effect above 260 °C may result from the evolution of carbon dioxide. Based on the presented DSC curves (Figure 8, Figure 9 and Figure 10), it can be stated that for all three types of fibers no significant differences in thermal properties were observed.

### 3.5. Porosity

The test confirming the above observations is the porosity of the obtained fibers. The results are shown in the table below (Table 2).

Observing the above table, we can conclude that the introduction of nano additives into the structure of the fibers causes the transformation of the morphological nature of the examined fibers in the direction of increasing the pore diameter. The largest pore diameter was characteristic for fibers with 7% TiO_2_ nano filler in their structure, which is expected due to the high share of TiO_2_ in the fiber structure.

### 3.6. Mechanical Properties

In order to determine which of the obtained fibers have the best strength properties, they were subjected to strength tests with unidirectional stretching. The table (Table 3) below presents the average values of the results obtained for a given research material.

Analyzing the table above, we can see that the fibers containing nano additive in their structure increase their strength properties. The fibers with 2% nano ZnO have the best properties. The introduction of titanium dioxide or zinc oxide into the spinning solution results in ordering the structure, which increases their strength.

### 3.7. Water Sorption at RH60 and Water Retention Value 

The reconstruction of the nature of the porous structure as a result of the incorporation of nano additives into the fiber structure has a beneficial effect on the moisture absorption capacity. The table (Table 4) below shows that fibers containing nanodilts metal oxides in their structure significantly improve their sorption and retention properties. In the case of sorption, fibers containing 2% nano ZnO in their structure had better properties, while fibers with TiO_2_ showed better retention properties.

### 3.8. Antimicrobial activity studies

Antimicrobial activity tests were carried out using our own nonwoven fabric made of obtained fibers. The research included quantitative determination of the antimicrobial effect of textiles using the AATCC 100-1998 “Antimicrobial Finishes of Textile Materials” method, while the following species of test microorganisms were used in the research:

Escherichia coli ATCC 10536 (gram negative rod);
Staphylococcus aureus ATCC 6538 (gram positive granuloma);
Aspergillus niger ATCC 16404 (molds).

The basic indicator of the biological activity of the tested nonwovens was the value of the reduction in the number of microorganisms after 24 h of incubation calculated according to formula:

(2) $$R=\frac{N_{0}-N_{t}}{N_{0}}*100\%$$

where:

N_0_ – number of microorganisms cultured on a non-woven control after 0 hours;
N_t_ – number of microorganisms grown on modified non-woven fabric after 24 h.

In addition, biocidal (*A*_b_) and biostatic (*A*_st_) activity values were calculated for the results obtained using the formulas below:

(3) $$A_{st}=\log N_{t}^{\prime }/N_{t}$$

where:

N_t_’- number of microorganisms grown on control nonwoven after 24 h;
N_t_ - number of microorganisms grown on modified non-woven fabric after 24 h.

(4) $$A_{b}=\log N_{0}/N_{t}$$

where:

N_0_—number of microorganisms grown on control nonwoven after 0 h;
N_t_—number of microorganisms grown on modified non-woven fabric after 24 h.

For the purposes of the research, the scale of material activity assessment determined in accordance with the guidelines of the EN 1276 and EN 1650 standards when determining the killing and static effect for fungi and bacteria was determined. Before the start of the study, the microorganisms were activated: bacterian TSB medium (Tryptic Soy Broth, Merck, Germany) at 37 ± 2 °C for 24–48 h; mushroom—MEB (Malt Extract Broth, Merck, Germany) at 27 ± 2 °C for 72 hours (molds). Results of the number of microorganisms on the tested nonwovens at t = 0 and t = 24 h are shown in the table (Table 5) below.

Analyzing the table above, we can see that the use of 2% nano Zinc Oxide resulted in a more effective reduction of the microorganisms used than was the case using 7% Titanium Oxide. The best reducing properties were obtained for fibers containing ZnO and TiO_2_ compared to Aspergillus Niger; the reduction of microorganisms was 82.22% and 76.11%, respectively, and the value of biocidal activity was 0.75 and 0.62, respectively. In contrast, the smallest bacterial reduction activity was obtained in relation to Staphylococcus aureus for both types of fibers tested. Titanium oxide also did not show reducing properties against Escherichia coli, while the fibers containing ZnO showed 67.31% ability to reduce this type of bacteria and the value of biocidal activity was 0.73. The best biostatic properties were observed for fibers with ZnO content for Escherichia coli, the value of the indicator of their biostatic activity was 1.47.

## 4. Conclusions

The conducted tests confirmed the presence of used nano additives in the fiber structure. The presence of nano additives increases the degree of crystallinity, e.g., for fibers with a 7% TiO2 content from 14.52% to 39.36% and up to 20.75% for fibers containing 2% ZnO. The change in the crystal structure may be associated with the nucleation effect of the crystal phase on the added nanoparticles. The presence of nano ZnO in an amount of 2% also results in an increase in specific strength from 15.8 cN/tex to 18.38 cN/tex and up to 16.88 cN/tex for fibers with a 7% TiO_2_ content. The conducted research also indicates that the presence of nanocomponents in the produced fibers does not cause significant changes in the thermal properties of the obtained nanocomposites. Based on the results of sorption and retention, it can be stated that the obtained fibers are hydrophilic, and the presence of a nano additive (SEM test) increases their porosity, thanks to which they can absorb a greater amount of water vapor, as well as show greater water retention, which is caused by increased defectiveness of the fiber surface due to the presence of nano additive. The tested alginate nonwovens containing the addition of nano TiO_2_ did not show antibacterial efficacy against the bacteria Escherichia coli and Staphylococcus aureus, while the alginate fibers containing nano ZnO showed the ability to reduce these bacteria at 67.31% and 18.74%, respectively. Much better results were obtained with analysis using Aspergillus Niger mold. For the non-woven fabric containing TiO_2_ modified fibers in its structure, antimicrobial efficiency was 76.11%, while for fibers having ZnO in its structure it was 82.22. Summing up the conducted tests, it can be said that among the tested nonwovens, nonwoven containing 2% ZnO was characterized by greater activity against the tested bacterial and mold species.

## Figures and Tables

**Table 1:** Comparison of the degree of crystallinity of the obtained fibers.

Sample	The Degree of Crystallinity (%)
Alginate fibers without nano additive	14.52
Alginate fibers with 2% ZnO	20.75
Alginate fibers with 7% TiO_2_	39.36

**Table 2:** Porosity of obtained fibers.

Sample	Total Pore Area (m^2^/g)	Average Pore Diameter (nm)
Alginate fibers without nano additive	0.100 ± 0.005	37300 ± 2000
Alginate fibers with 2% ZnO	0.077 ± 0.005	42200 ± 2100
Alginate fibers with 7% TiO_2_	0.055 ± 0.005	43500 ± 2120

**Table 3:** Mechanical properties of obtained fibers.

Sample	Tensile Strength (cN)	Elongation at break (%)	Specific Strength (cN/tex)
Alginate fibers without nano additive	1106.16 ± 178.46	4.39 ± 2.18	15.80 ± 2.49
Alginate fibers with 2% ZnO	1720.18 ± 69.37	3.62 ± 0.93	18.38 ± 1.68
Alginate fibers with 7% TiO_2_	1147.64 ± 93.80	3.15 ± 0.79	16.88 ± 1.31

**Table 4:** Fiber sorption and retention properties.

Sample	Sorption RH 60 (%)	Water Retention Value (%)
Alginate fibers without nano additive	21.42	81.63
Alginate fibers with 2% ZnO	41.04	89.52
Alginate fibers with 7% TiO_2_	30.07	95.46

**Table 5:** Bactericidal and bacteriostatic properties of obtained fibers.

Sample	Type of Microorganism	Reduction (%)	Biostatic Activity	Biocidal Activity
Alginate fibers without nano additive	*Escherichia coli*	-	-	-
	*Staphylococcus* *aureus*	-	-	-
	*Aspergillus niger*	-	-	-
Alginate fibers with 2% ZnO	*Escherichia coli*	67.31	1.47	0.73
	*Staphylococcus* *aureus*	18.74	0.51	0.09
	*Aspergillus niger*	82.22	0.37	0.75
Alginate fibers with 7% TiO_2_	*Escherichia coli*	-	0.57	0.46
	*Staphylococcus* *aureus*	-	0.26	-
	*Aspergillus niger*	76.11	0.24	0.62

“ - ”—no impact.
